# Loot

**Published:** 1868-03-15

**Authors:** 


### *LOOT.*

***Narceine.***

*Narceine* is coming into great fashion among the French, to replace *Morphia*. The dose generally given internally is from a sixth to half a grain. At the outset it diminishes the pulse, but subsequently accelerates the pulsations. It does not seem to produce constipation, but rather a free action of the bowels. It is said to retard menstruation. Dr. Eulenberg prefers it to any other narcotic, and gives it in neuralgia, in painful affections generally, and in articular diseases, iritis, cystitis, and orchitis, stating that it produces sleep, "which is soft, tranquil, uninterrupted, and followed by a quiet awakening." *Narceine* is reported to be preferable to *Morphia*, as a general rule, and to act effectually in those cases in which *Morphia* fails.—*St. Louis Medical Rep.*

***Arsenic in the Treatment of Pulmonary Phthisis.***

Dr. Moutard Martin, physician to the Hospital Beaujon, presented a paper to the Paris Academy of Medicine, upon the value of *Arsenic* in the treatment of pulmonary phthisis. The following conclusions of his memoir, we take from the *Gazette Hebdomadaire*:

1. Arsenical medication has a very positive action upon pulmonary phthisis.

2. That action is more efficacious in slow phthisis than in phthisis accompanied by fever.

3. Rapid phthisis and acute granular phthisis are not modified by arsenic.

4. In a certain number of cases, even in advanced phthisis with hectic, the general state of the patients is favorably modified, at least for a time, which time may be quite prolonged.

5. The modification of the local lesions is very slowly produced.

6. A certain number of cures should be attributed to arsenical medication, which would be more numerous if the patients did not too soon believe themselves cured, and if they had more perseverance.

7. To be efficacious, the treatment should be long-continued.

8. The *Arsenic* should be administered in extremely small doses.

9. Daily doses should not exceed two centigrammes. (Dr. Moutard Martin used granules of *Arsenious acid*, one milligramme each. He commences by one or two milligrammes, and gradually augments the dose according to individual tolerance, milligramme by milligramme.) [Full and diminishing doses are better. — Ed. C. M. J.]

10. *Arsenic* is better borne by patients slightly advanced, than by those who have reached the stage of excavation.

11. When doses do not exceed 15 milligrammes or 2 centigrammes, tolerance is, so to speak, indefinite.

12. The most manifest action of arsenical medication is corrobant, and secondarily, modifying of the pulmonary lesion. Yet, certain facts prove that *Arsenic* exerts a direct action upon the respiratory function; it may have an action upon the pulmonary tissue itself, and upon tubercle.

### *Intestinal Puncture in Tympanites.*

Under the advice of Dr. Foussagrives, intestinal puncture, as a last resource, has been several times practiced at Toulouse, on two patients suffering with tympanites. In the first case, the abdomen formed an immense mass; the patient was perfectly cyanosed and suffocating. On July 15th, M. Lafargue inserted an exploring trocar into the most distended part of the lower umbilical region. The gas escaped so violently as to extinguish a candle. The danger of asphyxia was thus removed; but the distension reappeared the next day, notwithstanding the use of ice and compression. Two fresh punctures were made in different places, and gave so much relief that the life of the poor patient was prolonged until July 20th, when he succumbed to his disorder without suffering from the other complication. The success and the harmlessness of the operation are still more evidenced in a second case, in which, notwithstanding all the means employed, Dr. Ressequet found the patient half asphyxiated. He made a puncture without any ulterior trouble, and five others were afterwards successively made, until the gases were naturally evacuated, and the patient cured. These cases give solid reasons for the practice of this method, so apparently dangerous, but so really harmless.—*L'Union Medicale.*

### *The Preparation of Pepsin.*

The following method of preparing *Pepsin*, was published by Dr. Beale, in his *Archives of Medicine:* "The mucous membrane of a perfectly fresh pig's stomach is carefully dissected from the muscular coat, and placed on a flat board. It is then cleaned with a sponge and a little water, and much of the mucus, remains of food, etc., carefully removed. With a back of a knife, or with an ivory paper knife, the surface is scraped very hard, in order to press the glands and squeeze out their contents. The viscid mucus thus obtained contains the pure gastric juice, with much epithelium from the glands and surface of the mucous membrane. It is spread out upon a piece of glass, so as to form a very thin layer, which is dried at a temperature of 100 degrees over hot water, or *in vacuo* over sulphuric acid. When dry, it is scraped from the glass, powdered, and kept in a stoppered bottle. A good digestive fluid may be made as follows: of the powder, 5 grains; strong *Hydrochloric acid*, 18 drops; water, 6 ounces. The fluid may be filtered easily, and forms a perfectly clear solution, very convenient for experiment. It may be taken with the salt, at a meal. The powder is devoid of smell, and has only a slightly salt taste. This powder undergoes no change, if kept perfectly dry. It contains the active principle of the gastric juice almost unaltered. Eight-tenths of a grain of this pepsin, with ten drops of dilute *Hydrochloric acid* and an ounce of distilled water, dissolve one hundred grains of hard-boiled white of egg in from twelve to twenty-four hours."

